# Supplementary material for: Traumatic injury compromises nucleocytoplasmic transport and leads to TDP-43 pathology
Source: eLife. 2021 Jun 1;10:e67587. doi: 10.7554/eLife.67587 (PMC8169113; doi:10.7554/eLife.67587)
Supplement: Transparent reporting form [file elife-67587-transrepform1.docx]

***eLife’s* transparent reporting form**

We encourage authors to provide detailed information *within their submission* to facilitate the interpretation and replication of experiments. Authors can upload supporting documentation to indicate the use of appropriate reporting guidelines for health-related research (see [EQUATOR Network](http://www.equator-network.org/%20)), life science research (see the [BioSharing Information Resource](https://biosharing.org/)), or the [ARRIVE guidelines](http://www.plosbiology.org/article/info:doi/10.1371/journal.pbio.1000412) for reporting work involving animal research. Where applicable, authors should refer to any relevant reporting standards documents in this form.

If you have any questions, please consult our Journal Policies and/or contact us: [editorial@elifesciences.org](mailto:editorial@elifesciences.org).

**Sample-size estimation**

- You should state whether an appropriate sample size was computed when the study was being designed
- You should state the statistical method of sample size computation and any required assumptions
- If no explicit power analysis was used, you should describe how you decided what sample (replicate) size (number) to use

Please outline where this information can be found within the submission (e.g., sections or figure legends), or explain why this information doesn’t apply to your submission:

For all data reported in this manuscript no sample size calculation was performed. However, for all Drosophila (western blot, immunofluorescence, behavioural, life-span, and drug testing), rat (immunohistochemical), and human (immunohistochemical, immunofluorescence, and ELISA) data collection, we followed previously published sample size (Anderson et al., 2018, Lanson et al., 2011; Daigle et al., 2013; Pandey et al., 2007; Zhang et al., 2015; Tamir et al., 2017; Sozda et al., 2010; Lajud et al., 2019; Wiesner et al., 2010; McKee et al., 2010; Cherry., et al 2016; Cherry et al., 2017) as a rational for sample size selection. In addition, all sample size used to acquire data are clearly reported in the figure legends of each panel.

**Replicates**

- You should report how often each experiment was performed
- You should include a definition of biological versus technical replication
- The data obtained should be provided and sufficient information should be provided to indicate the number of independent biological and/or technical replicates
- If you encountered any outliers, you should describe how these were handled
- Criteria for exclusion/inclusion of data should be clearly stated
- High-throughput sequence data should be uploaded before submission, with a private link for reviewers provided (these are available from both GEO and ArrayExpress)

Please outline where this information can be found within the submission (e.g., sections or figure legends), or explain why this information doesn’t apply to your submission:

All information regarding replicate (biological or technical) can be found in the method section as well as in the figure legend as n number. In addition, the amount of time an experiment was performed is reported in the method section. Also, all data point was reported, and none were excluded.

**Statistical reporting**

- Statistical analysis methods should be described and justified
- Raw data should be presented in figures whenever informative to do so (typically when N per group is less than 10)
- For each experiment, you should identify the statistical tests used, exact values of N, definitions of center, methods of multiple test correction, and dispersion and precision measures (e.g., mean, median, SD, SEM, confidence intervals; and, for the major substantive results, a measure of effect size (e.g., Pearson's r, Cohen's d)
- Report exact p-values wherever possible alongside the summary statistics and 95% confidence intervals. These should be reported for all key questions and not only when the p-value is less than 0.05.

Please outline where this information can be found within the submission (e.g., sections or figure legends), or explain why this information doesn’t apply to your submission:

Statistical analysis methods are all reported in the method section as well as the type of statistical methods and the precision measure used are reported for each figure in the figure legend. In general, all statistic method and type are listed in the figure legend.

(For large datasets, or papers with a very large number of statistical tests, you may upload a single table file with tests, Ns, etc., with reference to sections in the manuscript.)

**Group allocation**

- Indicate how samples were allocated into experimental groups (in the case of clinical studies, please specify allocation to treatment method); if randomization was used, please also state if restricted randomization was applied
- Indicate if masking was used during group allocation, data collection and/or data analysis

Please outline where this information can be found within the submission (e.g., sections or figure legends), or explain why this information doesn’t apply to your submission:

Sample were allocated into group base on the genotype of the animals (Drosophila) or whether they received trauma or not. Masking was not used.

**Additional data files (“source data”)**

- We encourage you to upload relevant additional data files, such as numerical data that are represented as a graph in a figure, or as a summary table
- Where provided, these should be in the most useful format, and they can be uploaded as “Source data” files linked to a main figure or table
- Include model definition files including the full list of parameters used
- Include code used for data analysis (e.g., R, MatLab)
- Avoid stating that data files are “available upon request”

Please indicate the figures or tables for which source data files have been provided:

Source files are listed for all figures in the manuscript as a single excel file. Each data set are label with the respected figures.
